# Application of a virtual and mixed reality-navigation system using commercially available devices to the lateral temporal bone resection

**DOI:** 10.1016/j.amsu.2021.103063

**Published:** 2021-11-16

**Authors:** Taku Ito, Yoshiyuki Kawashima, Ayame Yamazaki, Takeshi Tsutsumi

**Affiliations:** Department of Otolaryngology, Tokyo Medical and Dental University, Tokyo, Japan

**Keywords:** Virtual reality, Mixed reality, Surgical simulator, 3D hologram

## Abstract

**Background:**

Lateral temporal bone resection (LTBR) is performed for stage T1-2 external ear malignant tumors and requires spatial anatomical knowledge of the rare surgical field.

**Objective:**

This paper presents a novel virtual reality (VR) based surgical simulation and navigation system using only commercially available display device and an online software, to assist in the understanding of the anatomy pre and intraoperatively.

**Result and conclusion:**

VR model created by 3D Slicer modules and visualized on head mounted display enabled users to simulate and learn surgical techniques of a rare surgical case. 3D hologram through HoloLens assisted the surgeon in comprehending the spatial relationship between crucial vital structures and the pathological lesion during the operation. This platform does not require the users to possess specific programming skill or knowledge, and is therefore applicable in daily clinical usage.

## Highlights

● Lateral temporal bone resection (LTBR) is standard operative procedure for early-staged malignant tumors of external ear canals.
● However, many surgeons lack the opportunity to learn the surgical techniques because of its rarity.
● We report a usage of novel virtual reality based surgical simulation and navigation system for studying the anatomy and the operative steps in LTBR.
● 3D holograms with head-mounted display will provide revolutionary tool in assisting surgical planning, intraoperative referencing and navigation of otologic and skull base surgery.

## 1 Introduction

Lateral temporal bone resection (LTBR) is performed for stage T1-2 external ear malignant tumors according to Pittsburgh staging system [1,2]. LTBR requires en bloc removal of the external ear canal (EAC) including the tympanic membrane and malleus and incus. LTBR consists of several steps: disconnection of the ear canal from the temporal bone, such as complete mastoidectomy and extended facial recess approach, separation of superior ear canal from middle cranial fossa, and dissection of inferior ear canal [1,[3], [4], [5]]. These techniques are relatively common in middle ear surgeries, while temporomandibular joint disarticulation and dissection of medial tympanic ring and infratemporal fossa require difficult techniques to learn, because of its rarity. Head mounted displays (HMDs) have been recently utilized for the display of images in virtual reality (VR), completely immersing the user in a virtual environment, and obscuring the wearer's real-world field of view. Head-mounted display mixed reality (HMD-MR) technology further enables the projection of virtual 3D images into the user's real field of vision. These wearable device advancements and ever-evolving computer technologies can be powerful tools in the medical field, especially for facilitating therapeutic decision-making, such as through pre-operative planning of a surgical approach and through intra-operative surgical referencing [6,7].

In the present study, we report a usage of novel virtual reality (VR) based surgical simulation and navigation system for studying the anatomy and the operative steps in LTBR, using only commercially available display device and an online software. We fused key anatomical aspects: surface-rendered EAC, temporal bone, ossicles, jugular vein, carotid artery, facial nerve, and inner ear, based on a contrast enhanced CT scan. We believe that 3D holograms with HMD will provide revolutionary tool in assisting surgical planning, intraoperative referencing and navigation of otologic and skull base surgery. This report is approved by the Tokyo Medical and Dental University institutional review board.

## 2 Case

**Fig. 1:**
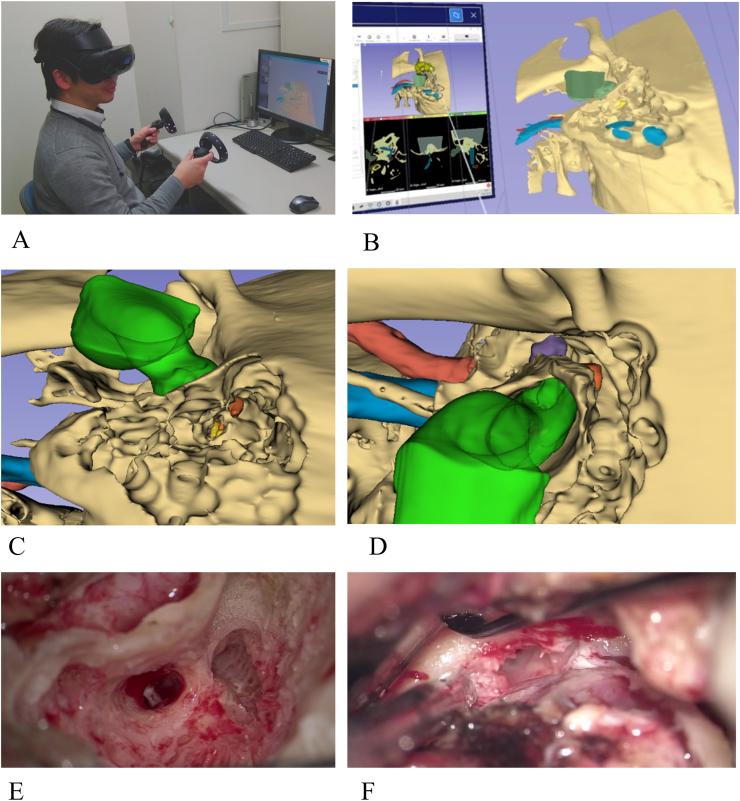
Surgical simulation of virtual 3D model using head-mounted display. After segmenting the individual anatomical parts, they were colored and overlayed on 2D (A) monitor and head mounted display (A, B). VR-based surgical simulators provide an immersive surgical experience that enhances the spatial awareness, speed, and dexterity necessary for surgery (A–D). 3D slicer module combined with head mounted display enable surgeons to simulate drilling and cutting of the bone using hand controllers. VR-based surgical views of posterior tympanotomy (C) and medial disarticulation (D) mimicked real surgical views seen through the microscope (E, F).

A virtual 3-D temporal bone model was reconstructed in a case of stage-T1 squamous cell carcinoma of EAC based on thin-slice CT, as described previously [8]. Briefly, we processed the patient's DICOM images by segmenting the EAC, temporal bone, ossicles, eustachian tube, jugular vein, and carotid artery using threshold segmentation technique. Facial nerve, cochlea, and semicircular canals were manually outlined by the surgeon on 3D Slicer software, a medical image analysis application (https://www.slicer.org/). For surgical simulation, five different modified bone segmentations were created from the original model by “Segment editor” module, according to each surgical step of mastoidectomy, posterior tympanotomy, separation of superior ear canal from middle fossa, dissection of inferior ear canal, and total resection. “Erase effect” was used to modify those models, which can remove the highlighted regions by clicking and/or dragging in 3D views from selected segment as if the surgeon drilled it out (Fig. 1B). Each process was viewed through the HMD of Windows Mixed Reality Headset (Fujitsu, Tokyo, Japan) (Fig. 1A). The surgeon could simulate and repeatedly practice the surgical procedures, preoperatively (Fig. 1C and D).

**Fig. 2:**
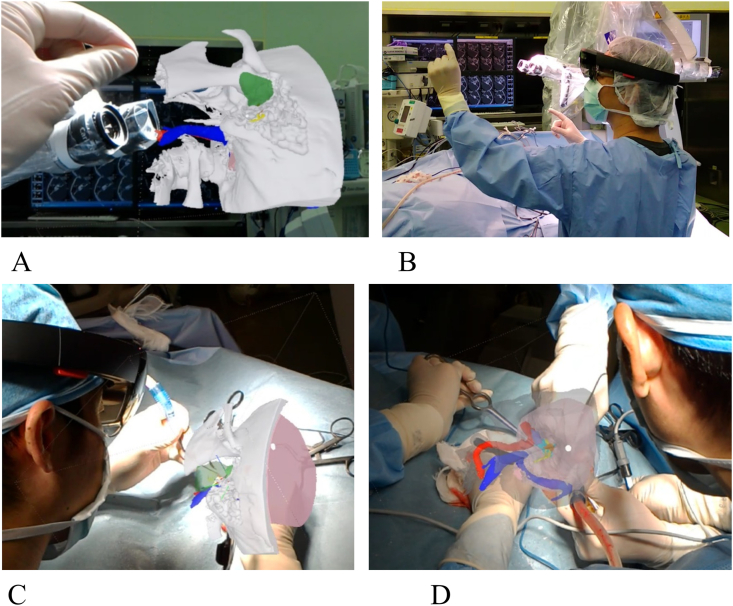
Intraoperative practical usages of Mixed reality. The surgeon wearing mixed reality headset intraoperatively. To keep the surgeon's hands sterile, the model can be manipulated through hand gesture (A, B). Superimposed 3D hologram on the real surgical field assist the surgeon to be aware of spatial relationship between crucial structures and the pathogenic lesion (C, D). Surgeon could rotate 3D hologram through HoloLens and change bone transparency (C, D) according to the surgical steps to recognize the three-dimensional relationships between the lesion (green), facial nerve (yellow), ossicles (orange), carotid artery (red), sigmoid sinus (blue), and middle cranial fossa (pink). (For interpretation of the references to color in this figure legend, the reader is referred to the Web version of this article.)

For intraoperative referencing and navigation, all 3D images were converted into STL files and uploaded on to “Holoeyes XR” website application (https://xr.holoeyes.jp/; Holoeyes, Tokyo, Japan). The 3D images had been automatically converted into a case-specific 3D hologram, which could be viewed through HoloLens (Microsoft, Seattle, WA, USA) (Fig. 2A and B) intraoperatively. The surgeon wearing the HoloLens could see the hologram-based surgical anatomy from a range of magnified view to the millimeter level inside the surgical field. It is also possible to change the surgeon's viewpoint, so that they can see not only inside the organ, but also view the surgical field from outside on various angles (Fig. 2C and D). Holoeyes XR is web application, which is available anywhere in the world. The surgeon can switch through the different surgical step models by means of a hand-gesture. Bone drilling was carried out while wearing HoloLens to ascertain the anatomic morphology of the temporal bone. 3D holographic images assisted and navigated the surgeon in identifying the antrum, facial recess, facial nerve, and middle cranial fossa. 3D holograms were helpful in establishing spatial anatomical comprehension of EAC, middle fossa, eustachian tube, and carotid artery. No intraoperative complications occurred.

## 3 Discussion

To perform temporal bone surgery safely and accurately, surgeons require a profound understanding of the 3D spatial relationships of critical structures such as tympanic membrane, ossicles, facial nerve, sigmoid sinus, middle cranial fossa, labyrinth, and carotid artery. Visualization of case-specific virtual 3D models created by 3D Slicer could generate different views from multiple angles and allow precise three-dimensional distance between those and the pathological lesion [8,9]. In this case, it provided useful information to the surgeons including the choice of drills, direction of facial recess, height of the middle cranial fossa, and the depth of the carotid artery.

3D Slicer is a free, cross-operating open-source extensible software that specializes in medical image visualization and computation [9]. 3D Slicer provides the users with the ability to generate digital anatomical 3D models without any specialized equipment or specific programming knowledge. One of the main advantages of 3D Slicer is that a variety of modules designed for different research applications are included in the software. “Segment Editor” module is a tool to delineate regions in the image and separate individual structures in the data set based on a patient CT scan. In this study, segmentations of the bony structures, carotid artery and jugular vein were performed automatically using threshold technique while the other structures were delineated semi-automatically or manually. It took less than 1 h to complete the tasks. Each segment was reconstructed together in the 3D space. Next, SlicerVirtualReality extension was utilized, which enables the user to interact with the 3D scene using virtual reality HMD [10,11]. This VR view was synchronized with the 3D Slicer scene. Hence, when we used “Erase” module to drill the mastoid in the 3D view, it was instantly reflected in the VR view, enabling the user to simulate mastoidectomy, posterior tympanotomy, and lateral temporal bone resection. Each process requires a certain level of diagnostic ability and surgical experience but not specialized computer skills.

Various surgical VR simulators have been developed, which allow surgeons to repeatedly practice a series of standardized surgical tasks. The Voxel-Man TempoSurg VR simulator is the commercially available temporal bone simulator and allows realistic handling of surgical drills by users. This platform consists of workstation, handpieces and foot pedal, therefore being immobile. In contrast, our current method requires only notebook PC and HMD, which is light enough to carry around and is less costly, therefore users can practice surgical procedure even at home. In addition, bone sawing procedure using this extension combined with haptic device was also reported, in the field of orthognathic surgery [10].

HMDs with installed mixed reality (MR) techniques have been used intra-operatively in surgery and showed promising results of reducing both the operating time and mental workload. MR experience can potentially improve surgical performance and can aid patients in understanding surgical procedures [[12], [13], [14]]. Several other VR-based navigation platforms were reported in spine surgery but could not compete with conventional navigation system in terms of accuracy [[15], [16], [17], [18], [19]]. Nhu et al. reported that a margin of error of about 10% was recorded in horizontal dimension, and up to 17% was reported in the z dimension for the indirect measurement of virtual objects through the HMD [20]. To improve the accuracy, several studies have been performed that highlighted further developments of the tools. Wu et al. utilized an AR system, which superimposes 3D anatomical models onto the patient using localization by superficial skin markers [21]. Koyachi et al. used registration markers attached to the oral splint [22]. Once HoloLens device recognized the registration marker attached to the splint, the operative field and the models were automatically aligned. While VR-based surgical navigation system is still under development, MR-based navigation surgery has some advantages. As aforementioned, it is easy to carry around and is relatively cheap. It also accommodates gesture controls, allowing surgeons to conduct surgery while continuing to look at the surgical field as HoloLens can overlay 3D hologram directly to the operative field. This may potentially contribute to shorter operative time.

In our study, different 3D bone models of serial surgical steps were created and shown on the HMD. Users could experience the surgical models preoperatively and refer them against the real surgical field intraoperatively. 3D holograms are based on a patient CT scan, but created by surgeon's subjective assessment on the MR-based navigation surgery. Therefore, the surgical simulation may increase the risk of overconfidence. Elaborative 3D models may not always guarantee good performance in live surgery, which requires experienced skills, careful observation and appropriate response to bleeding or unexpected complication. Despite this notice, the present study indicated that HMDs with VR and MR expansions could be introduced into daily clinical practice and can be useful for surgical training and planning, as well as anatomical referencing and navigation during surgery.

## 4 Conclusion

Case-specific VR-based surgical simulation allows the users to intuitively understand the positional relationships between organs, blood vessels and lesions within a patient's body, resulting in assistance for surgical planning and intraoperative management. Further study is warranted to evaluate the impact of VR-based surgical simulation and navigation with HMD on otological and lateral temporal bone surgery.

## Sources of funding

This work was supported by 10.13039/501100001691JSPS KAKENHI (Grant Number 17K11316) from the 10.13039/501100003478Ministry of Health, Labor, and Welfare of Japan and a research grant from Kao Melanin Workshop.

## Ethical approval

The protocols were approved by the Ethical Standards Committee of Tokyo Medical and Dental University (M2018-086-02) and are in accordance with the Declaration of Helsinki.

## Author contributions

T.I. designed the research, created 3D holograms, performed surgery and wrote the manuscript. Y.K. and T.T. assisted surgery and A.Y. provided critical feedback.

All authors in this paper declare that they have participated sufficiently in the conception and design of the work, and in the analysis of the data to take public responsibility for it.

All authors have read and approved this paper.

The principal investigator, T.I. indicates that he is prepared to take responsibility for the integrity of the content of the manuscript.

## Trial registry number

1. Name of the registry: 3D model assisted otologic guided surgery.

2. Unique Identifying number or registration ID: UMIN000045591.

3. Hyperlink to your specific registration (must be publicly accessible and will be checked): https://upload.umin.ac.jp/cgi-bin/ctr/ctr_view_reg.cgi?recptno=R000051473.

## Guarantor

Taku Ito.

## Consent

Informed consent was obtained for each participant.

## Provenance and peer review

Not commissioned, externally peer reviewed.

## Declaration of competing interest

The authors declare no conflicts of interest.
